# Genome-wide analysis of the TCP transcription factor family in mung bean and its dynamic regulatory network under salt stress

**DOI:** 10.3389/fpls.2025.1602810

**Published:** 2025-06-27

**Authors:** Zhi-Wei Wang, Guan Li, Ru-Zhi Li, Ru-Mei Tian, Min Liu, Xue Chen, Song Hou, Jiu-Yan Zhao, Yong-Yi Yang, Kun Xie, Na Qin, Longxin Wang, Lian-He Zhang, Kai-Hua Jia, Na-Na Li

**Affiliations:** ^1^ Agricultural College, Henan University of Science and Technology, Luoyang, China; ^2^ National Saline-Alkali Tolerant Crop Germplasm Resources Nursery (Dongying), Shandong Crop Germplasm Resources Bank, Institute of Crop Germplasm Resources, Shandong Academy of Agricultural Sciences, Jinan, China; ^3^ Legume Crops Research Institute, Weifang Academy of Agricultural Sciences, Weifang, China; ^4^ Department of Rehabilitation Medicine, The Second Affiliated Hospital of Shandong First Medical University, Taian, China; ^5^ School of Biological Science and Technology, University of Jinan, Jinan, China

**Keywords:** TCP, transcription factor, *Vigna radiata*, salt stress, co-expression network

## Abstract

The TCP gene family encodes plant-specific transcription factors that regulate plant growth, development, and stress responses. Although this gene family has been widely studied in various species, its function in mung bean (*Vigna radiata*) remains unclear. In this study, we identified 26 VrTCP genes, which were classified into two groups: Class I (PCF subfamily) and Class II (CYC/TB1 and CIN subfamilies). These family members likely function in the nucleus. VrTCP genes are unevenly distributed across chromosomes and are associated with gene duplication events. Their cis-regulatory elements are involved in plant growth, hormone signaling, and stress responses. Co-expression network analysis further supports these findings, identifying 1,304 genes co-expressed with VrTCPs, among which *VrTCP19*, *VrTCP10*, *VrTCP16*, and *VrTCP20* act as hub genes regulating hormone signaling and the MAPK pathway. Overall, VrTCP genes play a key role in salt stress responses, providing molecular insights that may facilitate the development of salt-tolerant mung bean varieties through molecular breeding. These findings also offer a foundation for future functional studies aimed at improving crop resilience under abiotic stress conditions.

## 1 Introduction

Transcription factors (TFs) are pivotal regulators of plant development and environmental responses, acting through DNA-binding domains (DBDs) to modulate gene expression by targeting specific cis-regulatory elements (Li et al., 2019). Among these, the plant-specific TCP family—named after TEOSINTE BRANCHED 1 (TB1) in maize, CYCLOIDEA (CYC) in Antirrhinum, and PROLIFERATING CELL FACTOR 1/2 (PCF1/2) in rice—has emerged as a crucial coordinator of diverse physiological processes (Cubas et al., 1999). Based on variations in the TCP domain, the family is divided into two major classes with distinct DNA-binding specificities and functional roles (Kosugi and Ohashi, 2002; Savadel et al., 2021; Zhang et al., 2021; Yu et al., 2022; Wu et al., 2023). Class I TCPs generally promote growth, while Class II TCPs often act as growth repressors involved in hormonal pathways and stress responses (Bai et al., 2012; Ortiz-Ramírez et al., 2016; Zheng et al., 2018).

The TCP transcription factor family is plant-specific and evolutionarily conserved, with gene copy numbers varying across species—24 in *Arabidopsis thaliana* (Nicolas and Cubas, 2016), 22 in rice (*Oryza sativa*) (Yao et al., 2007), 48 in banana (*Musa acuminata*) (Wang et al., 2022), 29 in *Phoebe bournei* (Lv et al., 2024), and 27 in sweet cherry (*Prunus avium*) (Dong et al., 2024). TCPs function as key regulators of plant development, orchestrating diverse processes such as leaf morphogenesis, lateral branching, floral organ patterning, and fruit ripening. For example, *TCP4* in *A. thaliana* regulates pistil development through the CRC–NGA module and downstream auxin-responsive pathways (Wang et al., 2024); *SlTCP12*, *SlTCP15*, and *SlTCP18* modulate fruit maturation in tomato (*Solanum lycopersicum*) (Danisman, 2016); *CsTCPs* in *Camellia sinensis* are involved in apical bud development and catechin biosynthesis (Yu et al., 2021); *LsTCP4* contributes to the morphological transition from the Salinas to Empire type in lettuce (*Lactuca sativa*) (Seki et al., 2020); and *PavTCP17* regulates floral bud dormancy in sweet cherry (Wen et al., 2023). In addition to their pivotal roles in development, TCPs have also been implicated in regulating plant responses to abiotic stresses, with growing evidence highlighting their involvement in stress adaptation. In rice, *OsTCP19* integrates developmental and stress signaling networks to coordinate plant adaptation under adverse conditions (Mukhopadhyay and Tyagi, 2015). Functional studies across species reveal similar regulatory potential: overexpression of *PeTCP10* or *PheTCP9* from Moso bamboo enhances salt tolerance in *A. thaliana* by promoting antioxidative activity and limiting Na^+^ accumulation (Liu et al., 2020; Xu et al., 2021); *BpTCP20* in *Betula platyphylla* improves drought and salt resistance via enhanced antioxidant enzyme function (Li et al., 2024); and *HrTCP20* in Platycladus orientalis confers drought resilience through modulation of jasmonic acid (JA) signaling (Yao et al., 2022).

Time-ordered gene co-expression network (TO-GCN) analysis has recently emerged as a powerful approach for resolving the temporal architecture of gene regulation in plants responding to environmental stimuli. By capturing stage-specific co-expression patterns, TO-GCN enables the reconstruction of transcriptional hierarchies and the inference of regulatory relationships between transcription factors and their target genes (Chang et al., 2019; Nie et al., 2022). Although this framework has primarily been applied to time-course transcriptome datasets—for example, revealing salt-responsive modules related to photosynthesis, osmotic regulation, flavonoid metabolism, and hormone signaling in *Populus* (Zhao et al., 2023)—its utility in concentration-dependent stress responses remains largely unexplored. In this study, we extend the application of TO-GCN to investigate transcriptional dynamics across a gradient of salt concentrations, offering new insights into how plants fine-tune gene regulatory networks in response to varying stress intensities.

Mung bean (*Vigna radiata*) is a fast-growing, protein-rich legume of major economic and nutritional importance, especially across tropical and subtropical regions (Hossen et al., 2021; Rane et al., 2021; Diatta et al., 2024). It serves as a critical dietary component in developing countries, offering affordable sources of high-quality protein, carbohydrates, folate, and iron (Hou et al., 2019). Yet, escalating soil salinity threatens mung bean production, impairing germination, vegetative growth, and reproductive success (Manasa et al., 2017; Alharby et al., 2019; Breria et al., 2020). This makes the development of salt-tolerant mung bean varieties an urgent priority. Despite the crop’s economic significance, the molecular mechanisms underlying mung bean’s response to salt stress remain poorly understood, with a lack of comprehensive research on its gene regulatory networks in saline environments. This gap in knowledge hinders efforts to breed salt-tolerant varieties, underlining the need for deeper investigation into the molecular bases of salt tolerance in mung bean.

Here, we present a systematic genome-wide analysis of the TCP gene family in mung bean, integrating bioinformatics, transcriptomic profiling, and stress-induced expression patterns. This study lays the groundwork for elucidating the transcriptional regulatory mechanisms of *VrTCP* genes by systematically analyzing their structural, functional, and regulatory characteristics under salt stress. These findings not only advance basic knowledge of stress-adaptive transcription factors in legumes but also identify candidate genes and molecular resources for breeding salt-tolerant mung bean varieties.

## 2 Materials and methods

### 2.1 Identification of VrTCP genes in mung bean

The mung bean genome used in this study is a high-quality Telomere-to-Telomere (T2T) genome previously assembled by our team (Jia et al., 2025). The genomes of *A. thaliana* and rice were downloaded from the Phytozome database (https://phytozome-next.Tgi.doe.gov/).To identify the VrTCP gene family, we first downloaded the Hidden Markov Model (HMM) profile for TCP TFs (Pfam ID: PF03634) from the Pfam protein family database (El-Gebali et al., 2018) and performed searches using the HMMER software (https://github.com/EddyRivasLab/hmmer) with default parameters. Next, *A. thaliana* TCP protein sequences were compared against mung bean protein sequences using BLAST, with the parameter “-evalue” set to 1e-5 and other settings left as default. BLAST results were filtered based on an identity threshold of 30%, and the filtered results were intersected with the Pfam search results. This process led to the identification of 26 VrTCP genes. These genes were renamed sequentially as *VrTCP1* to *VrTCP26* based on their chromosomal locations.

The conserved domains of all VrTCP proteins were verified using the Batch CD-Search tool (https://www.ncbi.nlm.nih.gov/Structure/bwrpsb/bwrpsb.cgi) with default parameters (Marchler-Bauer et al., 2016). The physicochemical properties of VrTCP proteins, including molecular weight, theoretical isoelectric point, instability index, aliphatic index, and grand average of hydropathicity, were predicted using the TBtools-II software (Chen et al., 2023). Finally, the subcellular localization of all VrTCP proteins was predicted using the WoLF PSORT website (https://wolfpsort.hgc.jp/).

### 2.2 Phylogenetic analysis

The amino acid sequences of VrTCP and AtTCP proteins were aligned using Muscle5 (v5.1) (Edgar, 2022) with default parameters. The resulting alignment was used to construct an unrooted maximum likelihood (ML) phylogenetic tree with FastTree (v2.1.11) (Price et al., 2010), applying the Jones-Taylor-Thornton (JTT) model, which is the default substitution model in FastTree. Branch length optimization and SH-like local support values were also set to default. An unrooted tree was chosen because no appropriate outgroup was available, and the focus was on illustrating the relative relationships among VrTCP and AtTCP proteins rather than inferring the direction of evolutionary change. The unrooted structure allows for a neutral representation of sequence similarity without assuming a specific evolutionary path. The unrooted tree was visualized in the Interactive Tree of Life (iTOL) (Letunic and Bork, 2021), where branch colors and styles were adjusted to distinguish different TCP protein groups.

### 2.3 Protein tertiary structure, motif, and gene structure analysis

The protein tertiary structures were predicted using the SWISS-MODEL website (https://swissmodel.expasy.org/), integrating models generated from the AlphaFold database to visualize structural features. Motif analysis was performed using the Multiple Em for Motif Elicitation (MEME) (Bailey et al., 2009) with the “Any Number of Repeats” (anr) mode, setting the number of motifs to 10 and the length range to 6–200. The input data consisted of protein amino acid sequences. Gene structure information and visualization based on the annotation file were analyzed using GSDS 2.0 (Hu et al., 2014).

### 2.4 Chromosomal localization, synteny analysis, and *cis*-regulatory element prediction

The chromosome locations of genes were determined based on the mung bean genome annotation file and visualized using MapChart software (Voorrips, 2002).

The synteny analysis includes both intra-species and inter-species components. For intra-species synteny, MCScanX (Wang et al., 2012) was used with default parameters to generate synteny files, and the results were visualized using Circos (Krzywinski et al., 2009). For inter-species synteny analysis, the file formats were then optimized using JCVI tools (v1.0.11) (Tang et al., 2008), removing duplicate information and matching the corresponding CDS and protein sequences. Pairwise comparisons of the genomes of *A. thaliana*, mung bean, and rice were conducted using the jcvi.compara.catalog module of JCVI tools, identifying syntenic blocks and extracting homologous gene pairs between species. The chromosome synteny relationships were visualized using the jcvi.graphics.karyotype module. Gene and sequence extraction and filtering were performed using SeqKit (v2.4.0) (Shen et al., 2016).

The upstream 2000 bp sequences of VrTCP genes were extracted as promoter regions using the -up-stream parameter of SeqKit software (Shen et al., 2016) for the identification and prediction of *cis*-regulatory elements (CREs). These sequences were then analyzed using PlantCARE (Lescot et al., 2002) to determine the positions and quantities of CREs. Functional filtering, statistical analysis, and visualization of the CREs were conducted using R (v4.2.3) with the heatmap and ggplot2 (https://github.com/tidyverse/ggplot2) packages. Finally, the distribution of CREs was visualized using the GSDS 2.0 platform (Hu et al., 2014).

### 2.5 Transcriptome data and expression of VrTCP genes

To investigate the mechanisms of VrTCP genes in response to salt stress in mung bean, seeds were first germinated on moistened filter paper placed in Petri dishes. After 3 days of germination, the seedlings were transferred to hydroponic boxes filled with deionized water without added nutrients, ensuring that any observed effects were solely due to the experimental treatments. All seedlings were grown under controlled conditions in a growth chamber: constant temperature of 26°C, relative humidity of 50%, a 16-hour light period (200 µmol photons m−² s−¹, 26°C), and an 8-hour dark period. After one week of cultivation in the hydroponic system, salt treatments were initiated by supplementing the hydroponic solution with different concentrations of NaCl: T0 (0 mM NaCl), T30 (30 mM NaCl), T60 (60 mM NaCl), T90 (90 mM NaCl), T120 (120 mM NaCl), and T150 (150 mM NaCl). Salt stress was imposed via the hydroponic solution rather than by foliar application. After 72 hours of salt treatment, fully expanded primary leaves were collected, immediately frozen in liquid nitrogen, and stored at −80°C for RNA extraction. Each treatment included three biological replicates.

Total RNA was isolated from leaf tissues using TRIzol reagent, and RNA quality and quantity were assessed using the Agilent 2100 Bioanalyzer. cDNA libraries were then constructed and subjected to high-throughput sequencing on the DNBSEQ platform. To ensure data quality, all RNA-seq data were filtered using Fastp (default parameters) (Chen et al., 2018). Clean reads were subsequently aligned to the mung bean reference genome using HISAT2 (version 2.1.0, default parameters) (Kim et al., 2019).Gene expression levels were quantified using FeatureCounts (Liao et al., 2014). Genes with extremely low expression, TPM (Transcripts Per Million) < 1, in all samples) were filtered out.

### 2.6 Co-expression network construction

The TO-GCN was constructed following the method described by Zhao et al (Zhao et al., 2023). to investigate co-expression relationships between TFs and non-TFs. Pearson correlation coefficients (PCCs) were calculated for TF and non-TF pairs, with a PCC threshold set at 0.9. Genes that exhibited peak expression under 0 mM NaCl treatment followed by downregulation with increasing NaCl concentrations were selected as bait genes. Genes co-expressed with VrTCP genes were identified based on PCC values and subsequently incorporated into the network. The hierarchical structure of the TO-GCN was generated using a breadth-first search algorithm, and the resulting regulatory network was visualized using Cytoscape (Shannon et al., 2003).

### 2.7 Enrichment analysis

Functional annotation of Gene Ontology (GO) and Kyoto Encyclopedia of Genes and Genomes (KEGG) terms was performed using eggNOG-mapper v2 (Cantalapiedra et al., 2021) against the eukaryotic database with default parameters. Enrichment analysis was conducted using the clusterProfiler package (Xu et al., 2024). Genes with TPM ≥ 1 in at least one sample were used as the background set. Significantly enriched GO terms and KEGG pathways were identified using a hypergeometric test with Benjamini–Hochberg correction, and those with an adjusted *P*-value < 0.05 were considered statistically significant. Data visualization was performed using ggplot2 (https://ggplot2.tidyverse.org/).

### 2.8 qPCR validation of VrTCP gene expression

To validate the transcriptome-based expression profiles of VrTCP genes, quantitative real-time PCR (qPCR) was performed. Total RNA was extracted from leaf tissues subjected to the same salt treatments described above using the FastPure Universal Plant Total RNA Isolation Kit (Vazyme, China), following the manufacturer’s protocol. RNA integrity and concentration were confirmed using the Agilent 2100 Bioanalyzer. First-strand cDNA was synthesized from 1 µg of total RNA using the HiScript^®^ III Reverse Transcriptase (Vazyme, China).

Gene-specific primers for selected VrTCP genes were designed using Primer-BLAST (https://www.ncbi.nlm.nih.gov/tools/primer-blast/) and synthesized commercially. Primer sequences are listed in **Supplementary Table 5**. qPCR was conducted using the SupRealQ Purple Universal SYBR qPCR Master Mix (Vazyme, China) on a ROCH Real-Time PCR System. The thermal cycling conditions were as follows: 95°C for 30 s, followed by 40 cycles of 95°C for 10 s and 60°C for 30 s. Each reaction was performed in triplicate using three independent biological replicates.

The mung bean *VrActin* gene was used as an internal reference. Relative gene expression levels were calculated using the 2^−ΔΔCt method. Statistical significance between treatments was determined by independent samples t-test, with differences considered significant at *p* < 0.05.

## 3 Results

### 3.1 Identification of TCP genes and analysis of protein characteristics in mung bean

Using the Pfam database (number: PF03634) and BLAST comparison with *A. thaliana*, a total of 26 TCP members were identified in mung bean, named VrTCP1 to VrTCP26 based on their chromosomal locations. The physicochemical properties of these proteins were also analyzed (**Supplementary Table 1**).

The isoelectric point (pI) of the proteins ranges from 6.26 (VrTCP23) to 10.07 (VrTCP24), with 6 proteins exhibiting weakly acidic pI values (pI < 7), while the remaining 20 proteins are alkaline (pI > 7). Except for VrTCP5, the other proteins in the family are considered unstable (Instability Index > 40), with values ranging from 36.73 (VrTCP5) to 68.65 (VrTCP21). The aliphatic index of VrTCP proteins ranges from 54.05 (VrTCP25) to 87.01 (VrTCP2), with an average of 65.93. Nine family members exhibit low thermal stability (AI < 60), while the other 18 members show moderate thermal stability (60 ≤ AI ≤ 90). All VrTCP proteins exhibit high hydrophilicity (GRAVY < 0), suggesting their favorable solubility in aqueous solutions and a tendency to interact with water molecules. Subcellular localization prediction shows that all VrTCP proteins are localized in the nucleus, suggesting that their primary regulatory functions occur within the nucleus.

Chromosomal localization analysis revealed that the 26 VrTCP genes are unevenly distributed across 10 chromosomes, with no VrTCP family genes found on chromosome 03 (**Supplementary Figure 1**; **Supplementary Table 1**). Chromosomes 08 and 10 have the highest number of VrTCP genes, with 4 genes each, specifically *VrTCP16*~*VrTCP19* on chromosome 08 and *VrTCP21~VrTCP24* on chromosome 10. Chromosome 09 has the fewest VrTCP genes, with only 1 gene (*VrTCP20*). Chromosomes 01, 02, 05, and 11 each contain 2 VrTCP genes, while chromosomes 04, 06, and 07 each contain 3 VrTCP genes.

### 3.2 Evolutionary analysis of VrTCP gene family

**Figure 1:**
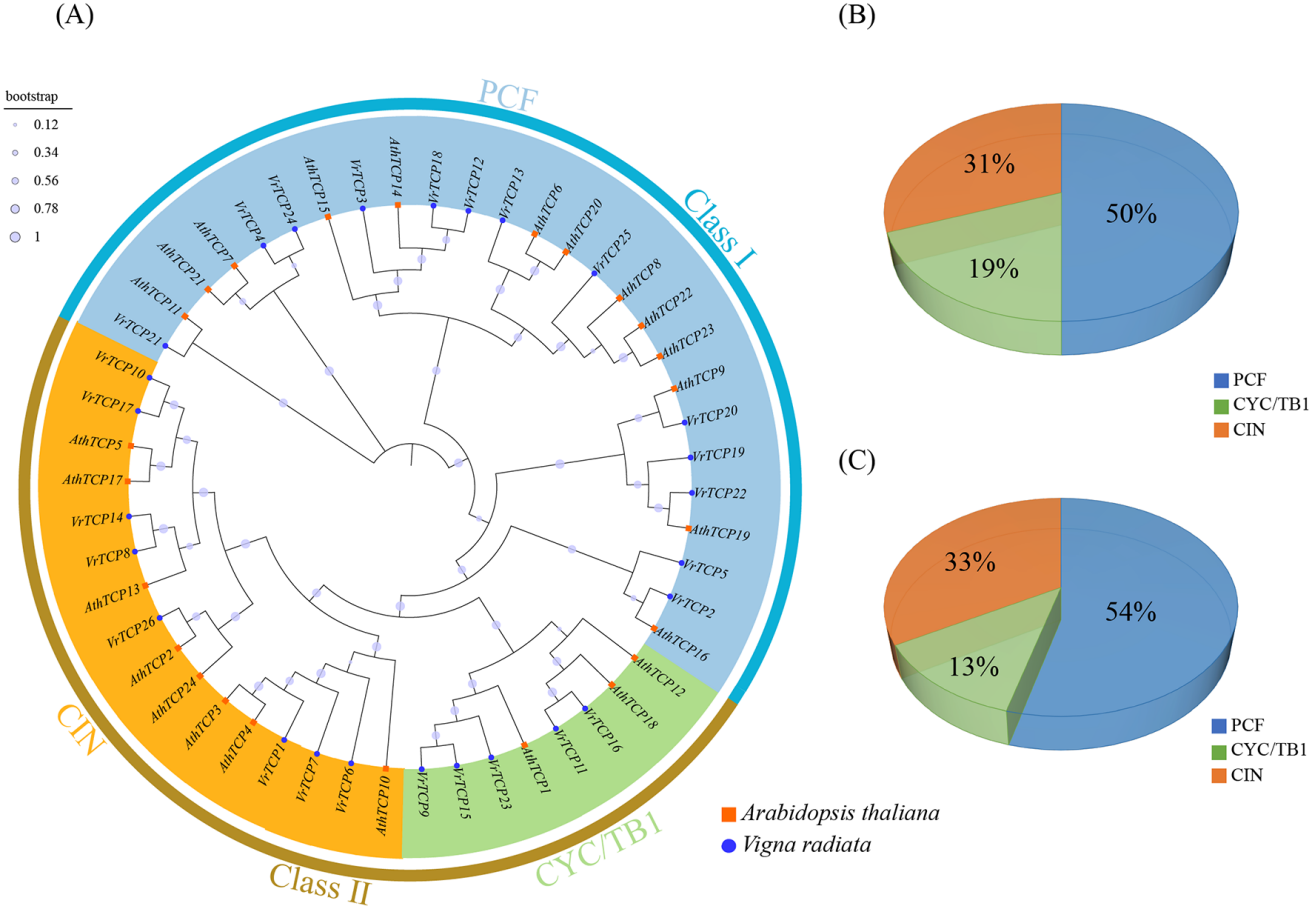
Phylogenetic analysis of TCP proteins. **(A)** Maximum likelihood tree of VrTCP and AtTCP proteins; blue circles indicate mung bean proteins, and orange squares indicate *A. thaliana* proteins. **(B, C)** Proportions of the three TCP subfamilies in mung bean and *A. thaliana*, respectively.

To investigate the evolutionary relationships of the VrTCP family, a phylogenetic tree was constructed using protein sequences from mung bean and *A. thaliana*, revealing that VrTCP proteins are classified into two classes and three subfamilies, with the PCF subfamily being the most abundant, suggesting that gene duplication and adaptive evolution may have contributed to their expansion and functional diversification (**Figure 1**). Based on multiple sequence alignment of 26 VrTCPs and 24 AtTCPs, the TCP proteins from the two species were classified into two classes and 3 subfamilies. Class I (PCF subfamily) contains 13 VrTCPs (50%) and 13 AtTCPs (54%). Class II is divided into two subgroups: the CYC/TB1 subfamily includes 5 VrTCPs (19%) and 3 AtTCPs (13%), while the CIN comprises 8 VrTCPs (31%) and 8 AtTCPs (33%). This distribution indicates that, although the number of genes in each subfamily varies between the two species, the proportion of TCP genes in the PCF subfamily is consistently higher than in the other two subfamilies (**Figure 1**).

The results suggest that the number of VrTCP proteins is higher than that of AtTCP proteins, which may reflect gene duplication events. Gene amplification not only provides mung bean with greater functional diversity but also may enhance its ability to respond to environmental stress and the complexity of its regulatory network, thereby conferring species-specific advantages during evolution.

### 3.3 Analysis of VrTCP protein sequences

This study analyzed the sequence and structural characteristics of 26 VrTCP proteins, comparing their conserved domain sequences with those of rice and *A. thaliana* TCP1 proteins. The results revealed four highly conserved motifs—basic, helix I, loop, and helix II—across species, with distinct differences between class I and class II TCPs. Class I TCPs lack four amino acid residues in the basic region, leading to different but related DNA binding sites (class I: GGNCCAC, class II: GTGGNCCC). Despite these differences, both classes maintain high overall conservation, which effectively distinguishes their subfamilies (**Supplementary Figure 2**).

Further analysis showed that the basic region exhibited the highest conservation, with residues such as lysine (K), aspartic acid (D), arginine (R), and histidine (H) being completely conserved across all TCP proteins. These residues may play key roles in protein function by participating in structural domains. The helix regions displayed a moderate level of conservation, while the loop regions were the least conserved, potentially reflecting differences in functional importance. These findings highlight the evolutionary conservation of TCP proteins and their potential functional divergence.

To investigate the structural characteristics of VrTCP proteins, AlphaFold3 was used to predict their three-dimensional structures. The structural analysis showed that α-helices exhibited the highest confidence scores, emphasizing their critical role in maintaining protein stability and function. These α-helices are likely essential for molecular recognition and active site formation. However, a substantial proportion of the predicted structures contained low-confidence regions, typical of low-complexity sequences lacking defined secondary structures (**Supplementary Figure 3**).

**Figure 2:**
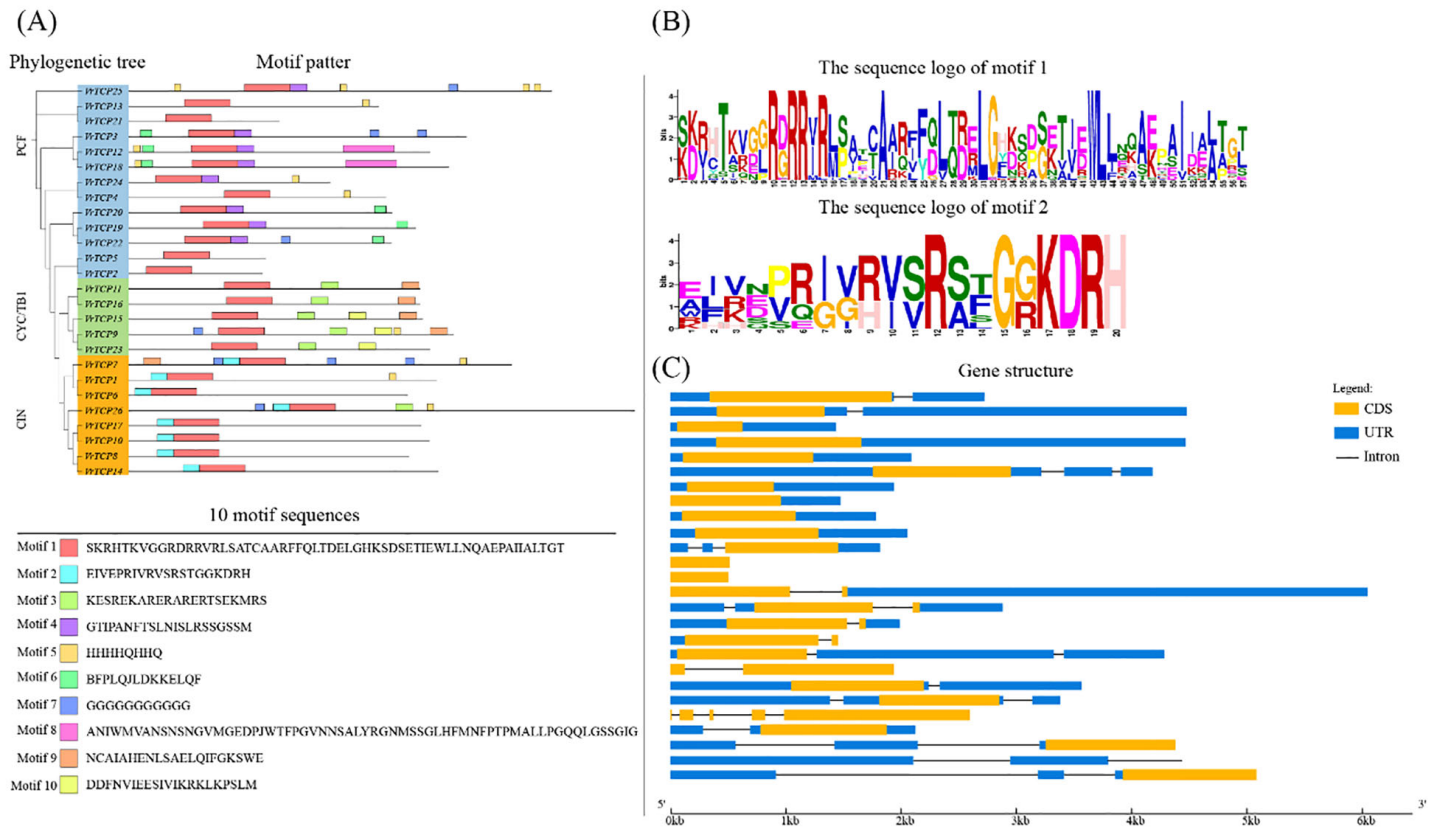
Protein motif composition and gene structure of VrTCP family members. **(A)** Phylogenetic tree and distribution of 10 conserved motifs (motifs 1–10, shown in different colors). **(B)** Sequence logos of motif 1 and motif 2, highlighting conserved amino acid residues. **(C)** Gene structures of VrTCP genes; orange boxes, exons; black lines, introns; blue boxes, UTRs.

We identified ten conserved motifs (motif 1–10) within VrTCP proteins (**Figure 2A**). Motif 1 was universally present across all VrTCP members, suggesting a fundamental role in protein function, while motif 2 was specific to the CIN subfamily and located at the N-terminus, distinguishing this subgroup. The sequence logos of motif 1 and motif 2 further illustrate their conserved amino acid patterns and subfamily-specific features (**Figure 2B**). Motif 4 was restricted to certain PCF subfamily members, indicating structural divergence among subfamilies. Notably, the conserved α-helices identified in the structural models overlapped with the positions of key motifs, further supporting their functional importance.

Gene structure analysis revealed variations among VrTCP proteins, with some exhibiting structural simplifications (**Figure 2C**). For instance, VrTCP2 and VrTCP5 contained only coding sequence (CDS) regions, while VrTCP26 lacked untranslated regions (UTRs), likely due to incomplete annotation rather than a biological feature. Additionally, VrTCP3, VrTCP4, VrTCP12, VrTCP19, VrTCP20, VrTCP21, and VrTCP24 lacked introns, which may represent an evolutionary adaptation to enhance transcriptional efficiency by reducing post-transcriptional processing complexity.

### 3.4 Synteny and evolutionary dynamics of the VrTCP proteins

**Figure 3:**
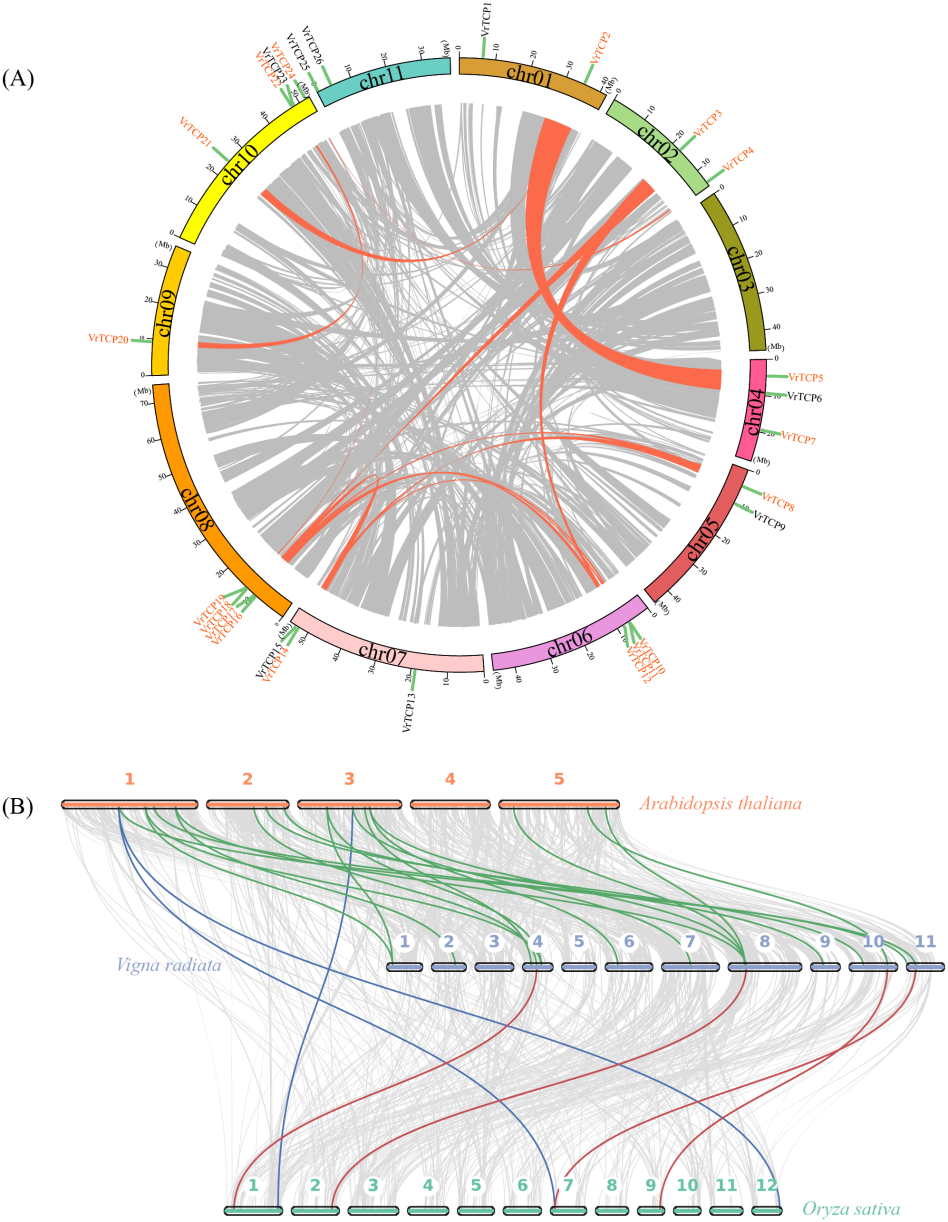
Comparative genomic and evolutionary analysis of TCP proteins. **(A)** Intraspecies collinearity of VrTCP proteins in mung bean; orange lines connect duplicated gene pairs across chromosomes. **(B)** Inter-species collinearity among *A*. *thaliana*, mung bean, and rice based on TCP protein sequences; colored lines denote orthologous relationships.

Collinearity analysis revealed extensive segmental duplications among VrTCP proteins, with no tandem duplications detected, suggesting strong evolutionary conservation (**Figure 3A**). A total of 15 segmental duplication pairs involving 18 VrTCP proteins were identified, including VrTCP2-VrTCP5, VrTCP2-VrTCP21, VrTCP3-VrTCP12, VrTCP3-VrTCP18, VrTCP4-VrTCP24, VrTCP8-VrTCP14, VrTCP8-VrTCP17, VrTCP10-VrTCP14, VrTCP10-VrTCP17, VrTCP12-VrTCP18, VrTCP11-VrTCP16, VrTCP14-VrTCP17, VrTCP19-VrTCP22, and VrTCP20-VrTCP22. Chromosome 08 contained the highest number of *TCP* genes and duplication events, while VrTCP17 and VrTCP18 participated in multiple duplication events. Notably, VrTCP17 was involved in three segmental duplication pairs (VrTCP8, VrTCP10, VrTCP14), while VrTCP18 participated in two (VrTCP3 and VrTCP12), suggesting their possible functional significance in gene expansion. Additionally, a unique duplication event was identified between VrTCP7 and Virad04G0090500, a non-TCP protein.

Cross-species collinearity analysis between mung bean, *A. thaliana*, and rice (**Figure 3B**) showed that mung bean shared 500 collinear protein pairs with *A. thaliana*, including 18 VrTCP proteins, while only 321 collinear protein pairs (4 VrTCP proteins) were identified between mung bean and rice. The lowest collinearity was observed between *A. thaliana* and rice, with 150 collinear protein pairs, including only 3 VrTCP proteins. These results indicate that TCP protein conservation is higher between mung bean and *A. thaliana* than between mung bean and rice.

### 3.5 Analysis of *cis*-regulatory elements in the promoter regions of VrTCP genes

**Figure 4:**
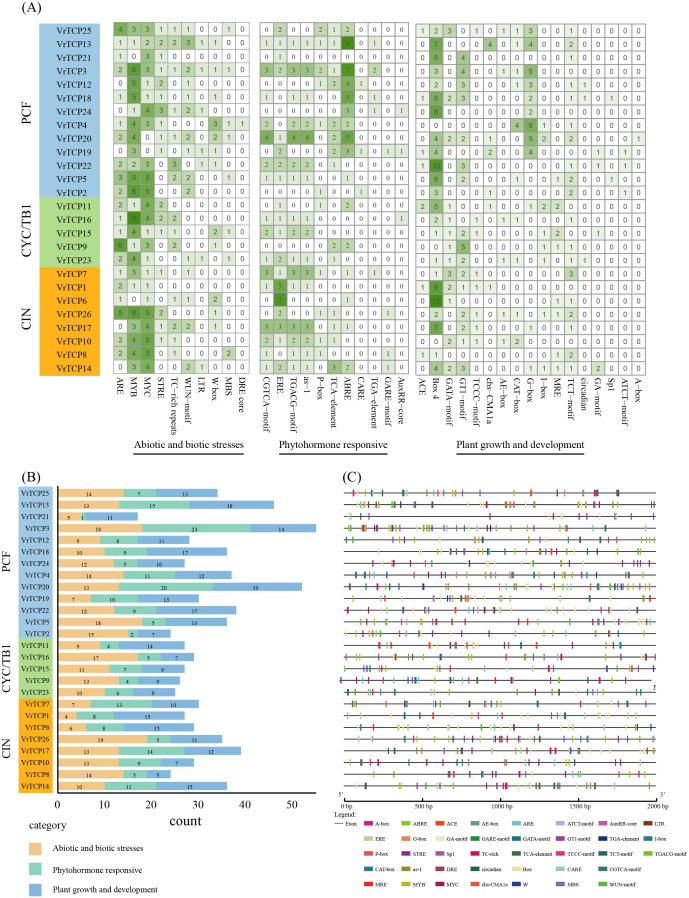
Analysis of CREs in VrTCP promoters. CREs were predicted from 2 kb upstream regions and classified into categories related to growth, hormone responses, and stress. **(A)** Heatmap of CRE abundance across VrTCP genes; deeper green indicates more elements. **(B)** Functional categorization of CREs across VrTCPs. **(C)** Distribution of CREs along promoter regions.

To elucidate the regulatory potential of VrTCP genes, CREs within 2000 bp promoter regions upstream of transcription start sites were systematically analyzed using the PlantCARE database. A total of 38 functionally annotated CREs were identified and categorized into three major groups: abiotic and biotic stress regulation (26.3%, 10 elements), phytohormone responses (28.9%, 11 elements) and plant growth and development (44.7%, 17 elements) (**Figure 4A**). The distribution of these elements showed significant heterogeneity among VrTCP members, with *VrTCP3* containing the highest number of CREs (55 elements) and *VrTCP21* the fewest (17 elements), suggesting functional diversification in their regulatory mechanisms (**Figure 4B**).

Significant variations were observed among functional categories of CREs. For abiotic and biotic stress regulation, CREs were most abundant in *VrTCP26* (19 elements) but minimal in *VrTCP1* (4 elements). Phytohormone-responsive elements were most prevalent in *VrTCP20* (20 elements) and nearly absent in *VrTCP21* (1 element). For plant growth and development-related *cis*-regulatory elements, *VrTCP20* exhibited the highest abundance (19 elements), in contrast to *VrTCP8* (5 elements) (**Figure 4B**). These disparities imply lineage-specific adaptation of promoter architectures to distinct physiological demands during mung bean growth and stress responses.

Notably, the distribution of CREs does not show a clear correlation with phylogenetic subfamily classification. VrTCP genes from different subfamilies exhibit similar functional CRE compositions, suggesting that their promoter regulatory features are primarily shaped by functional demands rather than strictly dictated by evolutionary lineage. This finding indicates that the *cis*-regulatory mechanisms of VrTCP genes may be more influenced by adaptive functional evolution rather than subfamily-specific divergence, challenging the hypothesis of subfamily-specific regulatory patterns.

### 3.6 Expression patterns and co-expression network analysis of VrTCP genes under salt stress

**Figure 5:**
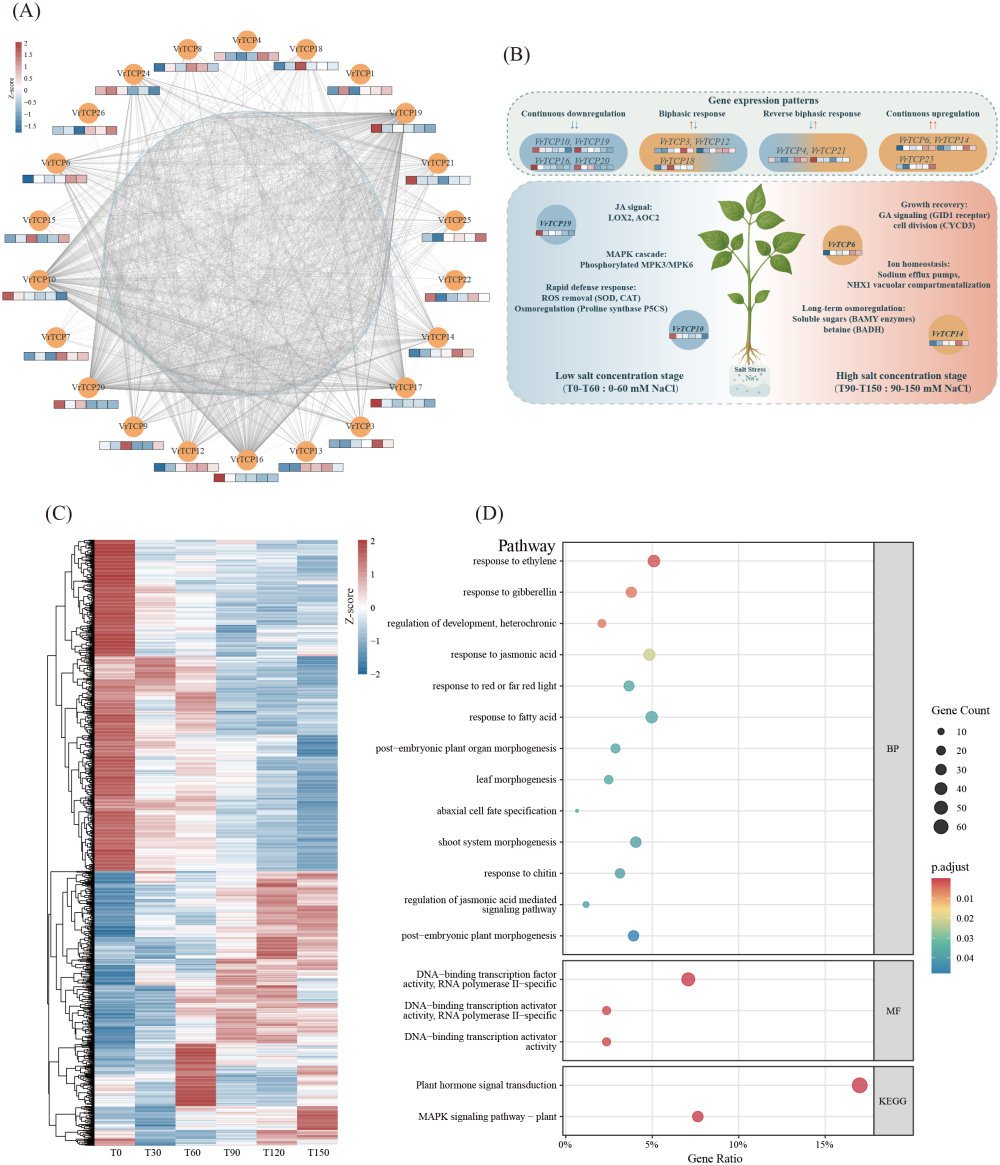
Integrated expression dynamics, co-expression networks, and functional enrichment of VrTCP genes under salt stress. **(A)** Co-expression network of VrTCP genes under salt stress. VrTCP genes are shown as orange nodes, with mini heatmaps below each node representing normalized expression levels (TPM) across different NaCl concentrations. Light blue nodes indicate co-expressed functional genes, and grey lines represent co-expression links. **(B)** Expression dynamics of VrTCP genes across a gradient of NaCl concentrations. VrTCPs were grouped into four expression patterns: continuous downregulation (*VrTCP10*, *VrTCP19*, *VrTCP16*, *VrTCP20*), biphasic response (*VrTCP3*, *VrTCP12*, *VrTCP18*), reverse biphasic response (*VrTCP4*, *VrTCP21*), and continuous upregulation (*VrTCP6*, *VrTCP14*, *VrTCP25*). These patterns correspond to distinct roles in ROS detoxification (e.g., SOD, CAT), osmotic adjustment (e.g., P5CS, BADH), hormone signaling (e.g., JA, GA), and ion homeostasis (e.g., NHX1, Na⁺ efflux), delineating VrTCP-mediated transcriptional regulation under low (T0–T60) and high (T90–T150) salt conditions. **(C)** Heatmap showing expression profiles of co-expressed genes. **(D)** GO and KEGG enrichment analysis of genes co-expressed with VrTCPs, highlighting functional pathways involved in salt stress adaptation.

To explore the potential role of VrTCP genes in salt stress responses, we utilized transcriptome data previously obtained from 18 mung bean seedling samples treated with different NaCl concentrations (T0–T150) for 72 hours. A total of 893,214,900 raw reads were generated, and after quality filtering, 893,105,466 clean reads were retained. Quality metrics were high across all samples, with Q20 and Q30 values exceeding 95%, and mapping ratios above 98% (**Supplementary Table 2**). Gene expression levels were subsequently calculated in TPM based on clean, mapped reads. These data were used to construct a TO-GCN focused on VrTCP genes and their co-expressed partners, revealing dynamic transcriptional responses under increasing NaCl concentrations (**Figure 5A**). Among the 26 identified VrTCP genes, 22 were expressed in mung bean leaves. Notably, *VrTCP1*, *VrTCP7*, *VrTCP14*, and *VrTCP26* exhibited higher expression levels, while *VrTCP9* and *VrTCP16* showed relatively low expression (**Supplementary Table 3**). To validate the transcriptome-based expression patterns, 12 VrTCP genes showing distinct expression trends were selected for qPCR analysis. The qPCR results were generally consistent with the RNA-seq data, confirming the reliability of transcriptome-derived expression profiles (**Supplementary Figure 4B**; **Supplementary Table 6**).

As NaCl concentration increased, distinct expression patterns were observed among the genes. For instance, *VrTCP6*, *VrTCP14*, and *VrTCP25* displayed increased expression with rising NaCl concentrations, suggesting theirz potential roles as positive regulators in the salt stress response. Conversely, *VrTCP19*, *VrTCP24*, *VrTCP16*, *VrTCP10*, and *VrTCP17* showed progressively decreased expression levels, indicating that these genes might be suppressed under elevated salt conditions. Additionally, *VrTCP3*, *VrTCP12*, and *VrTCP18* exhibited a biphasic pattern, initially upregulated at lower NaCl concentrations and subsequently downregulated at higher concentrations. This pattern suggests these genes may play roles in the early phase of the salt stress response, potentially activating specific pathways to mitigate initial damage. In contrast, *VrTCP4* and *VrTCP21* exhibited the opposite expression trend, with initial downregulation at lower NaCl concentrations, followed by upregulation at higher concentrations (**Figure 5A, B**).

Co-expression network analysis identified 1,304 genes with significant co-expression relationships with VrTCP genes (**Figure 5A**). Among them, *VrTCP19* (degree = 320), *VrTCP10* (degree = 227), *VrTCP16* (degree = 274), and *VrTCP20* (degree = 262) had the highest degrees in the network, indicating that these genes may play central regulatory roles in the co-expression network. Notably, *VrTCP19*, *VrTCP10*, *VrTCP16*, and *VrTCP20* all exhibited a gradual decrease in expression with increasing NaCl concentrations. This trend suggests that these genes may play significant roles during the early or mid-stages of salt stress and might be suppressed under higher salt concentrations, possibly as part of a feedback regulation mechanism in stress response.

Furthermore, these co-expressed genes showed distinct concentration-specific expression patterns, with some genes exhibiting high expression only at specific NaCl concentrations (**Figure 5C**). GO and KEGG enrichment analysis revealed that these genes were significantly enriched in pathways related to response to JA, response to gibberellin, response to ethylene, DNA-binding transcription activator activity, RNA polymerase II-specific, MAPK signaling pathway – plant, and Plant hormone signal transduction (**Figure 5D**; **Supplementary Table 4**).

## 4 Discussion

### 4.1 Expansion and diversification of the VrTCP gene family in mung bean

The 26 VrTCP genes exhibit an uneven chromosomal distribution, with a marked enrichment on chromosomes 8 and 10 (**Supplementary Figure 1**). This biased distribution pattern appears to be shaped predominantly by segmental duplication events rather than tandem duplications, suggesting that genomic constraints—such as dosage sensitivity and selective pressure to avoid local clustering—may influence the organization of transcription factor families (Hurles, 2004; Francis et al., 2016). The biased chromosomal distribution could reflect functional compartmentalization or co-regulation potential in specific genomic regions. For instance, clusters on Chr08 and Chr10 may harbor stress-responsive modules, potentially facilitating coordinated regulation during salt stress. The stronger collinearity with Arabidopsis than rice (**Figure 3B**) further supports a dicot-specific evolutionary trajectory (Sun et al., 2023).

Structural modeling of VrTCP proteins using AlphaFold3 revealed conserved α-helical domains characteristic of the TCP domain, supporting their roles in DNA binding and protein–protein interactions (**Supplementary Figure 3**). In particular, Class II members possess a four-amino-acid insertion within the TCP domain (**Supplementary Figure 2**), distinguishing them structurally from Class I proteins; this feature has been associated with altered DNA-binding specificity and potential regulatory roles under stress conditions (De Paolo et al., 2015; Ding et al., 2019). Additionally, motif 2—predominantly found in CIN subfamily members—corresponds to regions involved in leaf morphogenesis and growth control (Manassero et al., 2013), implying that VrTCP structural divergence may underpin functional specialization during abiotic stress adaptation.

### 4.2 Regulatory complexity revealed by promoter analysis and subcellular localization

Promoter CRE analysis indicated substantial regulatory diversity among VrTCP genes (**Figure 4**). Growth-related elements dominated promoters such as *VrTCP20*, while stress-responsive elements were enriched in *VrTCP26*, suggesting subfunctionalization into growth-promoting and stress-adaptive modules (Rath et al., 2022; Spears et al., 2022). This pattern echoes findings in wheat TCPs under biotic stresses (Li et al., 2022) and underscores the regulatory plasticity that may facilitate adaptive responses to fluctuating environments.

Consistently, all VrTCP proteins were predicted to localize in the nucleus (**Supplementary Table 1**), supporting their canonical role as transcriptional regulators (Leng et al., 2019). Nuclear residency is essential for modulating transcriptional networks involved in stress responses, development, and hormonal signaling, suggesting that spatial compartmentalization contributes to the functional versatility of TCP TFs (Challabathula and Bartels, 2013). The promoter and localization data highlight regulatory modularity among VrTCPs, offering molecular entry points for engineering stress-adaptive expression patterns.

### 4.3 Dynamic VrTCP gene expression patterns under salt stress and implications for functional specialization

Transcriptome analysis under salt stress revealed highly dynamic and gene-specific expression patterns (**Figure 5B**). *VrTCP6* and *VrTCP14* were strongly upregulated with increasing NaCl concentrations, suggesting that they may act as positive regulators of salt tolerance by promoting osmotic adjustment and enhancing ROS scavenging, mechanisms critical for salt adaptation as previously demonstrated for *OsTCP19* in rice (Mukhopadhyay and Tyagi, 2015). Conversely, *VrTCP19* and *VrTCP24* were progressively downregulated, indicating possible trade-offs between growth and stress resilience (Wang et al., 2020). Given that TCP TFs are broadly implicated in regulating osmotic balance and ROS detoxification (Mukhopadhyay and Tyagi, 2015; Xu et al., 2021), *VrTCP6* and *VrTCP14* are strong candidates for orchestrating these stress-adaptive responses in mung bean.

Interestingly, *VrTCP4* and *VrTCP21* exhibited delayed induction, suggesting involvement in late-phase salt adaptation processes such as ion homeostasis and long-term osmoregulation (Xu et al., 2021). These complex expression trajectories point to a phased regulatory model where distinct VrTCPs orchestrate early defensive responses, metabolic reprogramming, and long-term acclimation under salt stress.

### 4.4 VrTCP hub genes integrate hormonal and MAPK signaling to regulate salt stress responses

*VrTCP19*, *VrTCP10*, *VrTCP16*, and *VrTCP20* emerged as central regulatory hubs within the co-expression network (**Figure 5**), with functional enrichment pointing to their involvement in JA, gibberellin, and ethylene signaling pathways, as well as MAPK signaling cascades. These pathways represent core components of plant stress perception and transcriptional reprogramming, underscoring the potential role of these VrTCP genes in coordinating hormonal crosstalk and downstream adaptive responses (Mukhopadhyay and Tyagi, 2015; Xu et al., 2021). The central positioning of VrTCP hub genes within the network suggests that they may coordinate complex signaling pathways that balance growth and stress responses, a role consistent with the known function of TCP TFs in integrating hormonal crosstalk (Spears et al., 2022). Notably, MAPK cascades regulate both immediate defense activation and long-term adaptation by modulating gene expression, protein stability, and metabolic pathways (Daldoul et al., 2022), raising the possibility that VrTCPs act upstream or in parallel with MAPK-mediated responses during salt stress.

The transcriptional profiles of the hub genes further support this model: *VrTCP19*, *VrTCP10*, *VrTCP16*, and *VrTCP20* all exhibited a gradual downregulation with increasing NaCl concentrations, suggesting that their early activation and subsequent suppression may reflect a feedback regulatory mechanism to optimize resource allocation under prolonged stress. Comparisons across species reinforce these findings. In *A. thaliana*, *PeTCP10* enhances salt tolerance by promoting antioxidative defenses and limiting Na^+^ accumulation (Liu et al., 2020; Xu et al., 2021), while *BpTCP20* overexpression in *Betula platyphylla* improves drought and salt resistance through increased antioxidant enzyme activities (Li et al., 2024) Similarly, *HrTCP20* in *Platycladus orientalis* strengthens drought tolerance via modulation of JA signaling (Yao et al., 2022) a pathway also enriched among VrTCP co-expressed genes.

Interestingly, *VrTCP3*, *VrTCP12*, and *VrTCP18* exhibited a biphasic expression pattern, characterized by early induction followed by repression at higher salt concentrations. Such dynamics are reminiscent of the early, transient activation of JA-mediated defense signaling, which must later be attenuated to prevent trade-offs with growth. Conversely, *VrTCP4* and *VrTCP21* displayed the opposite trend, suggesting that different VrTCP members may have evolved specialized roles to fine-tune the temporal phases of stress adaptation. Supporting this idea, *VuTCP9* in *Vigna unguiculata* modulates stomatal development and delays senescence under stress conditions, contributing to enhanced drought and salt tolerance (Mishra et al., 2022). In maize, *ZmTB1* plays a central regulatory role by directly binding to the promoter of the HD-ZIP transcription factor *ZmGTT*, thereby activating ABA signaling to suppress lateral branching and enhance drought resistance (Viola et al., 2023). Under salt stress, *AtTCP1* accelerates flowering in *A. thaliana*, contributing to early escape from adverse conditions (Viola et al., 2023).These phased expression profiles underscore the temporal coordination of salt stress responses, providing candidate genes for stage-specific functional studies and breeding.

Taken together, these findings suggest that VrTCP hub genes play a pivotal role at the intersection of hormonal and MAPK signaling networks, regulating gene expression to balance immediate stress responses with long-term growth adaptation. This positions the VrTCP family as a promising target for genetic engineering, molecular breeding, and marker-assisted selection strategies aimed at enhancing salt stress resilience in mung bean and other legume crops. Such integration of VrTCPs within key signaling networks emphasizes their central regulatory roles and supports their use in multi-pathway stress resilience engineering.

### 4.5 Implications and future perspectives

This study provides the first genome-wide characterization of the TCP gene family in mung bean, revealing their structural features, regulatory potential and salt-responsive expression dynamics. The identification of key VrTCP genes involved in hormonal and MAPK signaling highlights their central role in coordinating growth and stress responses (Mukhopadhyay and Tyagi, 2015; Danisman, 2016; Liu et al., 2020; Xu et al., 2021; Wang et al., 2024). These findings offer a valuable genetic resource for functional studies and lay the groundwork for molecular breeding strategies aimed at improving salt tolerance in mung bean and other legume crops.

## 5 Conclusions

This study presents the first comprehensive characterization of the TCP transcription factor family in mung bean, identifying 26 VrTCP genes classified into Class I (PCF) and Class II (CYC/TB1 and CIN) subfamilies. These genes display diverse and dynamic expression patterns under salt stress, with *VrTCP6*, *VrTCP14*, and *VrTCP25* showing consistent upregulation, suggesting positive regulatory roles in salt tolerance. Co-expression network analysis highlighted *VrTCP19*, *VrTCP10*, *VrTCP16*, and *VrTCP20* as central hub genes involved in hormone signaling and MAPK pathways, underscoring their potential in coordinating growth-defense trade-offs. These findings deepen our understanding of the functional roles of TCP genes in legume stress responses and provide valuable candidates for future functional validation. Moreover, they offer molecular targets for the genetic improvement of salt tolerance in mung bean, and a foundation for cross-species exploration of stress-resilient regulatory networks in legumes.

## Data Availability

The genome-wide sequences and annotation data reported in this study have been deposited in the Genome Sequence Archive (GSA). The relevant data can be accessed via the following link: https://ngdc.cncb.ac.cn/gsa/, under the accession number PRJCA021300. The CDS sequences (Vra.TCP.cds.fasta) and protein sequences (Vra.TCP.pep.fasta) of the VrTCP family used in this study have been uploaded to the Figshare database. The data can be accessed via the following link: https://doi.org/10.6084/m9.figshare.28023824.v1. RNA-seq datasets from treatments with different NaCl concentrations and from treatments at different time points are publicly available under CNSA (the CNGB Sequence Archive) project numbers CNP0006168.
