# Supplementary material for: Genome-wide analysis of the TCP transcription factor family in mung bean and its dynamic regulatory network under salt stress
Source: Front Plant Sci. 2025 Jun 27;16:1602810. doi: 10.3389/fpls.2025.1602810 (PMC12247535; doi:10.3389/fpls.2025.1602810)
Supplement: Supplementary file 1 [file SupplementaryFile1.docx]

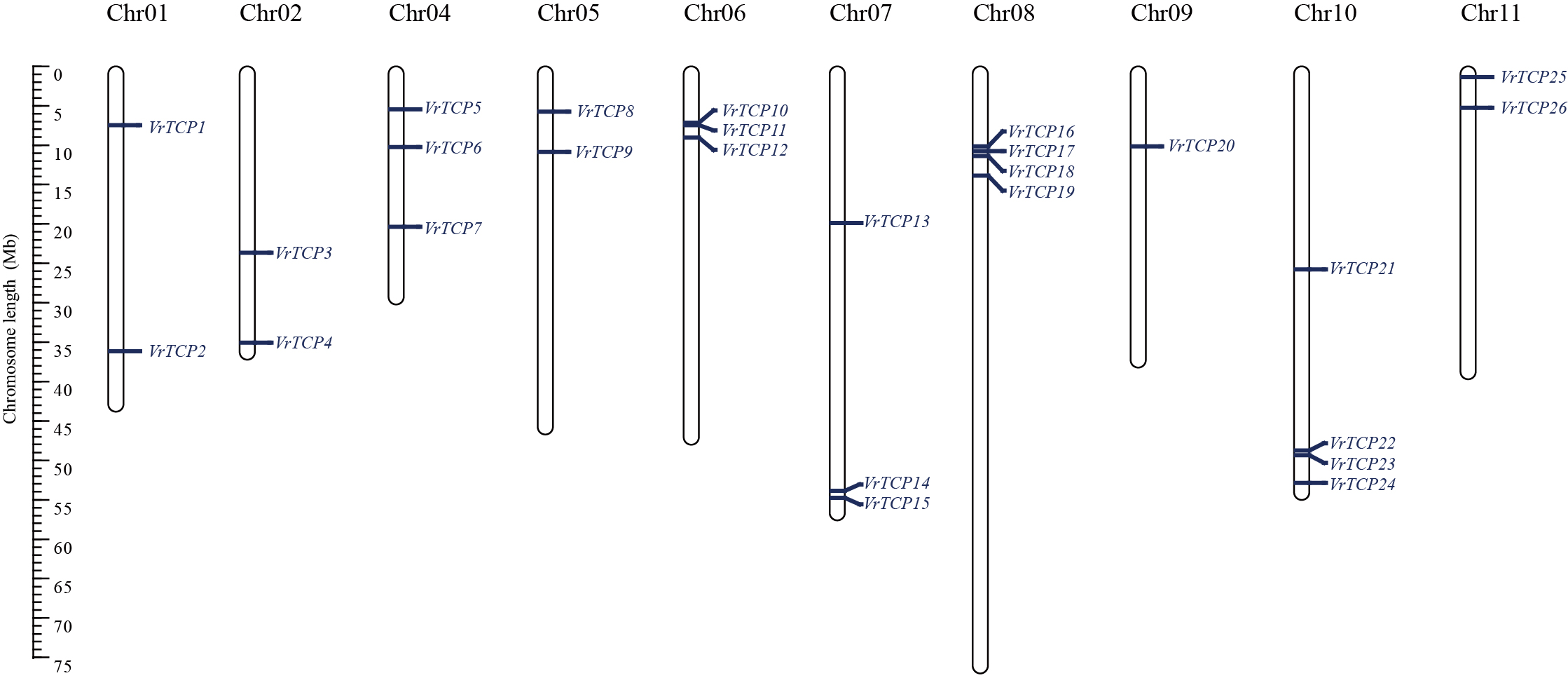


**Supplementary Fig. 1** **Chromosomal localization analysis of the *VrTCP* gene family in mung bean.** Chr01 to Chr11 correspond to mung bean chromosomes 1 through 11, while *VrTCP1* to *VrTCP26* indicate the precise localization of TCP gene family members on these chromosomes. The scale displayed on the left of each chromosome represents its length in megabases (Mb).


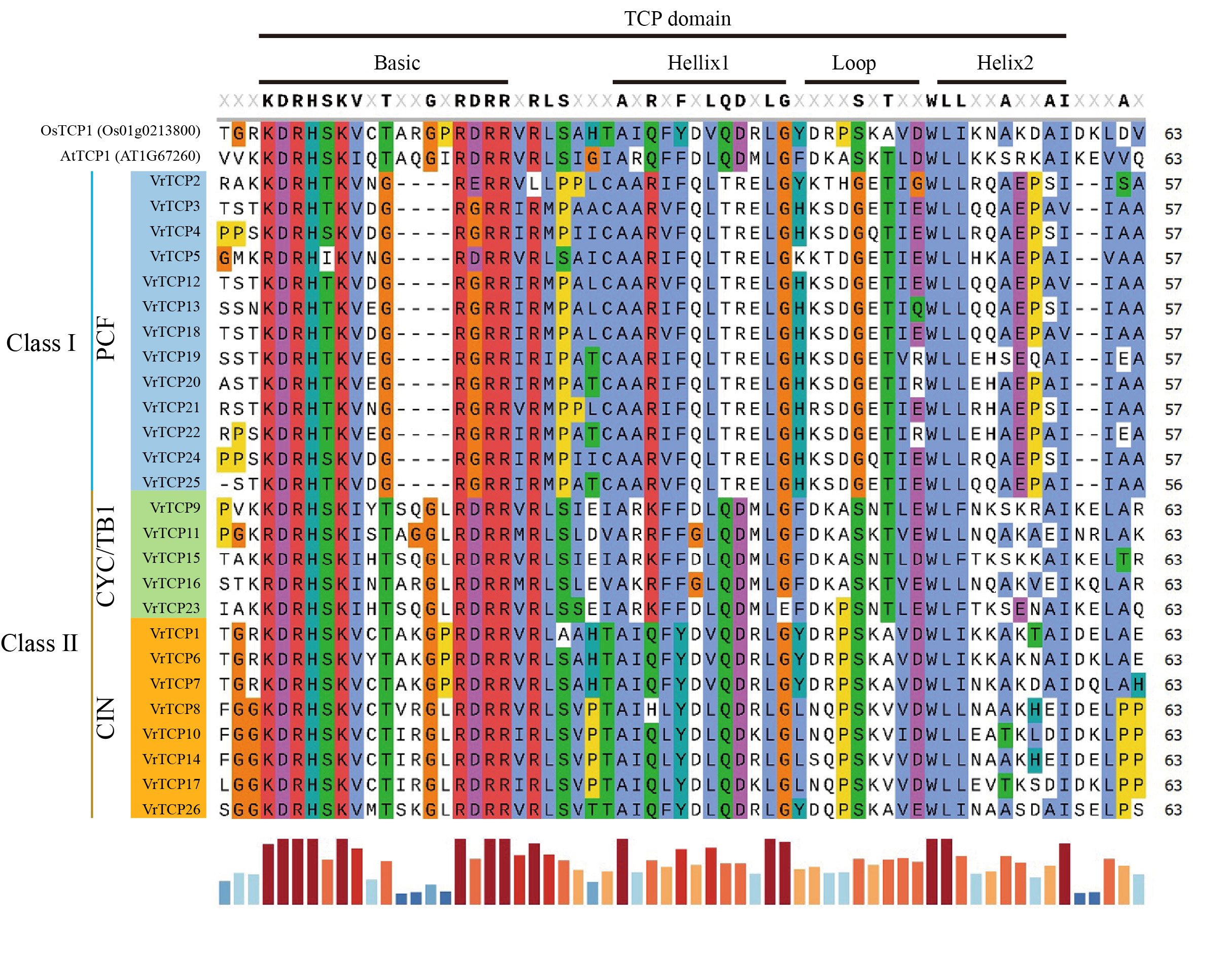


**Supplementary Fig. 2 Multiple sequence alignment of TCP protein domains.** OsTCP1 represents the rice TCP protein, and AtTCP1 represents the A. thaliana TCP protein. The bar chart at the bottom indicates the level of sequence conservation, with taller red bars representing higher conservation and shorter blue bars representing lower conservation.


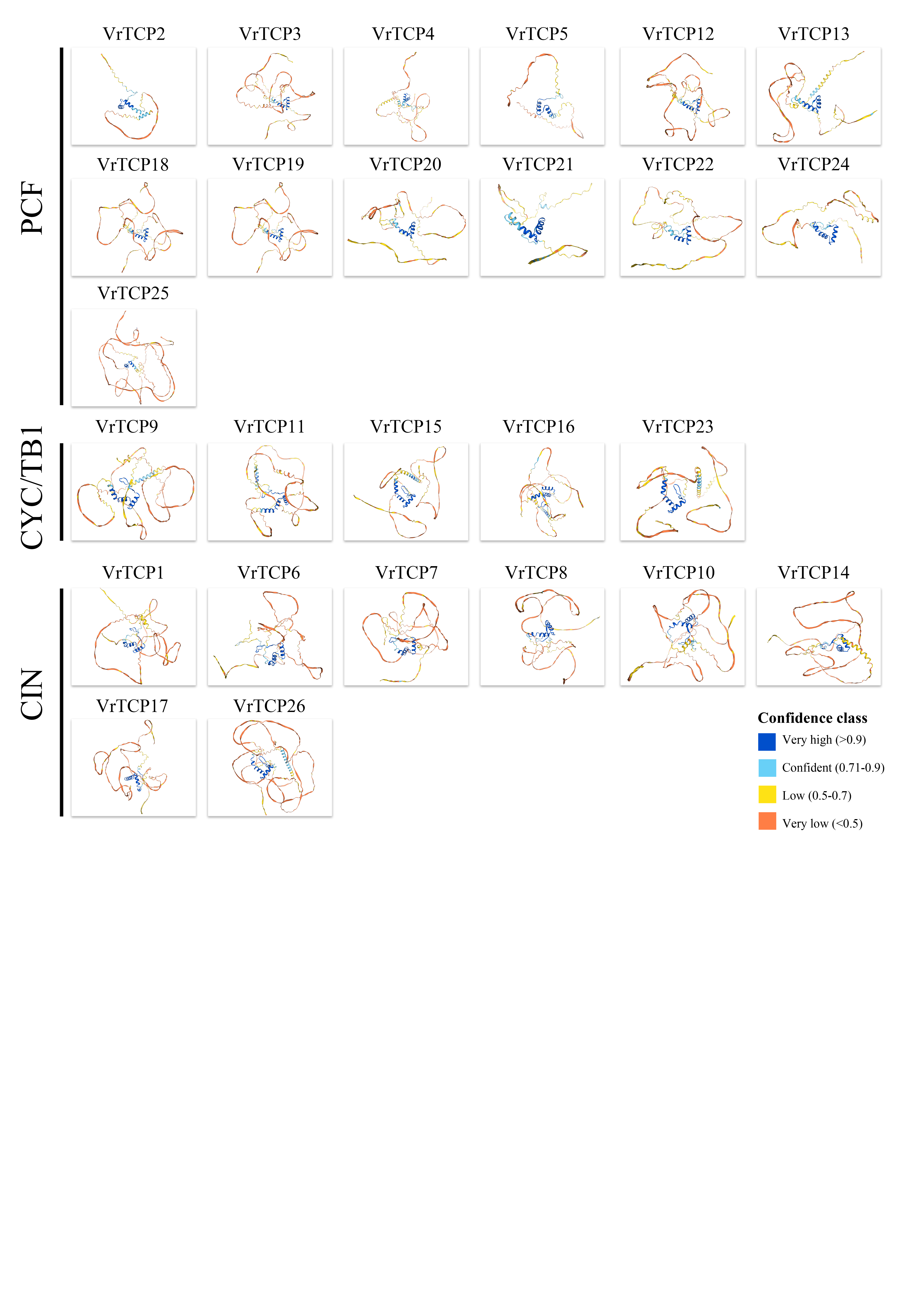


**Supplementary Fig. 3 Structural analysis of VrTCP proteins.** The left panel shows the classification of three subfamilies, with different colors indicating the confidence levels of protein three-dimensional structures. The bottom-right corner provides a legend for the four confidence levels, where blue represents the highest confidence and orange represents the lowest.


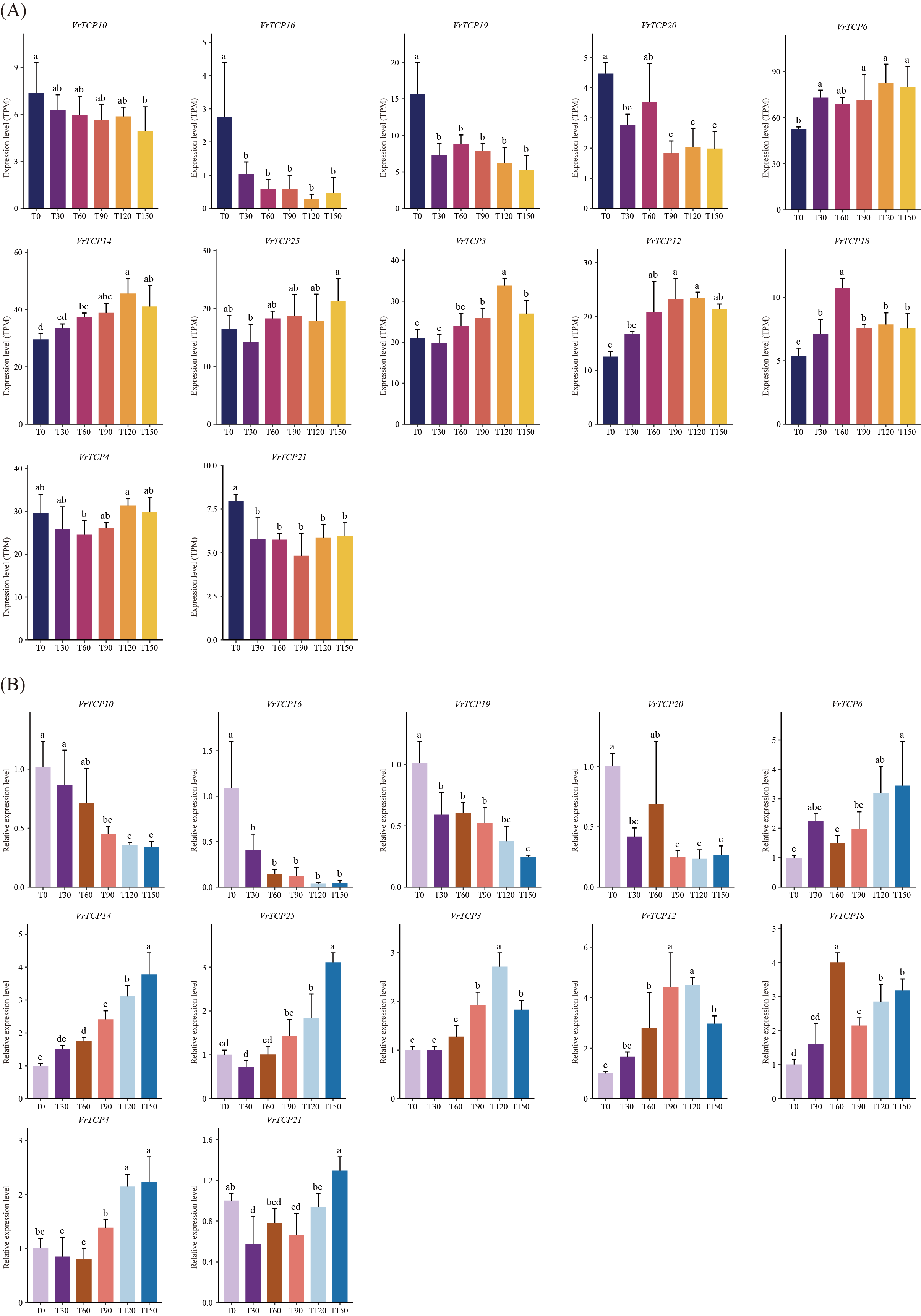


**Supplementary Fig. 4 Expression levels of VrTCP genes under different NaCl treatments.** (**A**) TPM-based expression levels of VrTCP genes under varying NaCl concentrations (T0–T150) from transcriptome data; (**B**) Relative expression levels validated by qPCR under corresponding treatments. Different lowercase letters indicate significant differences between treatments (T-test, *p* < 0.05).
